# Model-based unsupervised learning informs metformin-induced cell-migration inhibition through an AMPK-independent mechanism in breast cancer

**DOI:** 10.18632/oncotarget.16109

**Published:** 2017-03-10

**Authors:** Arjun P. Athreya, Krishna R. Kalari, Junmei Cairns, Alan J. Gaglio, Quin F. Wills, Nifang Niu, Richard Weinshilboum, Ravishankar K. Iyer, Liewei Wang

**Affiliations:** ^1^ Department of Electrical and Computer Engineering, University of Illinois at Urbana-Champaign, Champaign, IL, USA; ^2^ Department of Health Sciences Research, Mayo Clinic, Rochester, MN, USA; ^3^ Department of Molecular Pharmacology and Experimental Therapeutics, Mayo Clinic, Rochester, MN, USA; ^4^ Department of Mechanical Science and Engineering, University of Illinois at Urbana-Champaign, Champaign, IL, USA; ^5^ Wellcome Trust Centre for Human Genetics, University of Oxford, Oxford, UK; ^6^ Department of Pathology, University of Chicago, Chicago, IL, USA; ^7^ Weatherall Institute of Molecular Medicine, University of Oxford, Oxford, UK

**Keywords:** single cell, RNA-seq, breast cancer, metformin, unsupervised learning

## Abstract

We demonstrate that model-based unsupervised learning can uniquely discriminate single-cell subpopulations by their gene expression distributions, which in turn allow us to identify specific genes for focused functional studies. This method was applied to MDA-MB-231 breast cancer cells treated with the antidiabetic drug metformin, which is being repurposed for treatment of triple-negative breast cancer. Unsupervised learning identified a cluster of metformin-treated cells characterized by a significant suppression of 230 genes (*p-value* < 2E-16). This analysis corroborates known studies of metformin action: a) pathway analysis indicated known mechanisms related to metformin action, including the citric acid (TCA) cycle, oxidative phosphorylation, and mitochondrial dysfunction (*p*-value < 1E-9); b) 70% of these 230 genes were functionally implicated in metformin response; c) among remaining lesser functionally-studied genes for metformin-response was *CDC42*, down-regulated in breast cancer treated with metformin. However, CDC42's mechanisms in metformin response remained unclear. Our functional studies showed that *CDC42* was involved in metformin-induced inhibition of cell proliferation and cell migration mediated through an AMPK-independent mechanism. Our results points to 230 genes that might serve as metformin response signatures, which needs to be tested in patients treated with metformin and, further investigation of *CDC42* and AMPK-independence's role in metformin's anticancer mechanisms.

## INTRODUCTION

The emergence of high-throughput, single-cell RNA sequencing (scRNA-seq) has allowed investigators for the first time to determine levels of gene expression in hundreds of individual cells simultaneously [1]. In comparison to conventional RNA-seq, which provides an aggregate view of the cells in a tissue sample, scRNA-seq can simultaneously measure the expression level of the entire genome in all of the individual cells of the sample, allowing the characterization of all cell types and states present [1]. In this work we utilized the benefits of scRNA-seq to gain insights into metformin's molecular mechanisms in MDA-MB231 triple-negative breast cancer cells. Triple negative is the molecular subtype of breast cancer for which no highly effective targeted therapy currently exists [2]. Metformin is the most commonly used drug for reducing hyperglycemia in patients with type 2 diabetes [3], but is being repurposed for the prevention and treatment of cancer.

Evidence from *in vitro* and retrospective studies [4] suggests that metformin inhibits the growth of triple-negative breast cancer. Multiple mechanisms, including 5′-adenosine monophosphate-activated protein kinase AMPK-dependent and AMPK-independent mechanisms, have been suggested for the metformin effect in cancer treatment [5, 6]. However, the therapeutic effect of metformin in the treatment and prevention of TNBC remains unclear [7, 8], and there are no pharmacogenomic biomarkers for selecting responsive patients.

Our first preliminary analysis of homogenous MDA-MB-231 triple-negative breast cancer cells without metformin treatment demonstrated that distribution of gene expression in a cell was best described by a combination of distributions (mixtures). Next, we observed that metformin response is not uniform across all cells, because we found some cells whose distributions of gene expressions were altered differently. To further investigate this non-uniform response to metformin, we used mixture-model-based single-cell analysis (MiMoSA) [9], driven by mixture-model-based unsupervised learning, to infer single-cell subpopulations (clusters of cells) based on differences in their distributions, which can be used to drive focused functional studies. We used unsupervised learning in this work because of the lack of prior knowledge on gene expression distribution that characterizes metformin's response in triple-negative breast cancer.

To identify cells with altered gene expression distributions, MiMoSA inferred three clusters of cells, and in one of them, we observed a group of 230 genes that were significantly down-regulated (*p-value* < 0.0006) during metformin treatment which was sufficient to pursue with bioinformatics approaches such as pathway analysis. Several enriched metabolic pathways associated with metformin response such as the citric acid (TCA) cycle and respiratory electron transport, oxidative phosphorylation, mitochondrial dysfunction were also associated with 230 these genes. In the 230 genes on these mentioned pathways, nearly 70% of the genes had multiple functional evidence of anti-cancer mechanisms and offered little novelty in helping us understand metformin's mechanisms in triple-negative breast cancer [10, 11]. Remaining genes with lesser functional evidence comprised 24 genes. Included among these 24 genes was *CDC42*. *CDC42* is known for its effect on cell proliferation and cell migration. It has been shown to be involved in the metformin effect on neuroblastoma, and has been found to be significantly down-regulated in breast cancer patients treated with metformin [12, 13]. However, mechanisms by which *CDC42* might influence metformin response in breast cancer remain unknown. Therefore, we performed functional characterization of *CDC42* in the context of its role in metformin response in TNBC. Our functional studies found that *CDC42* was involved in metformin-induced inhibition of cell proliferation and cell migration mediated through an AMPK-independent mechanism, a novel mechanism for metformin's anti-metastatic action.

This work highlights the benefits of scRNA-seq and the ability of model-based unsupervised learning to identify biologically significant, yet subtle effects of metformin via the suppression of 230 genes in only 6 cells.

## RESULTS

### Sequencing data characteristics

**Figure 1:**
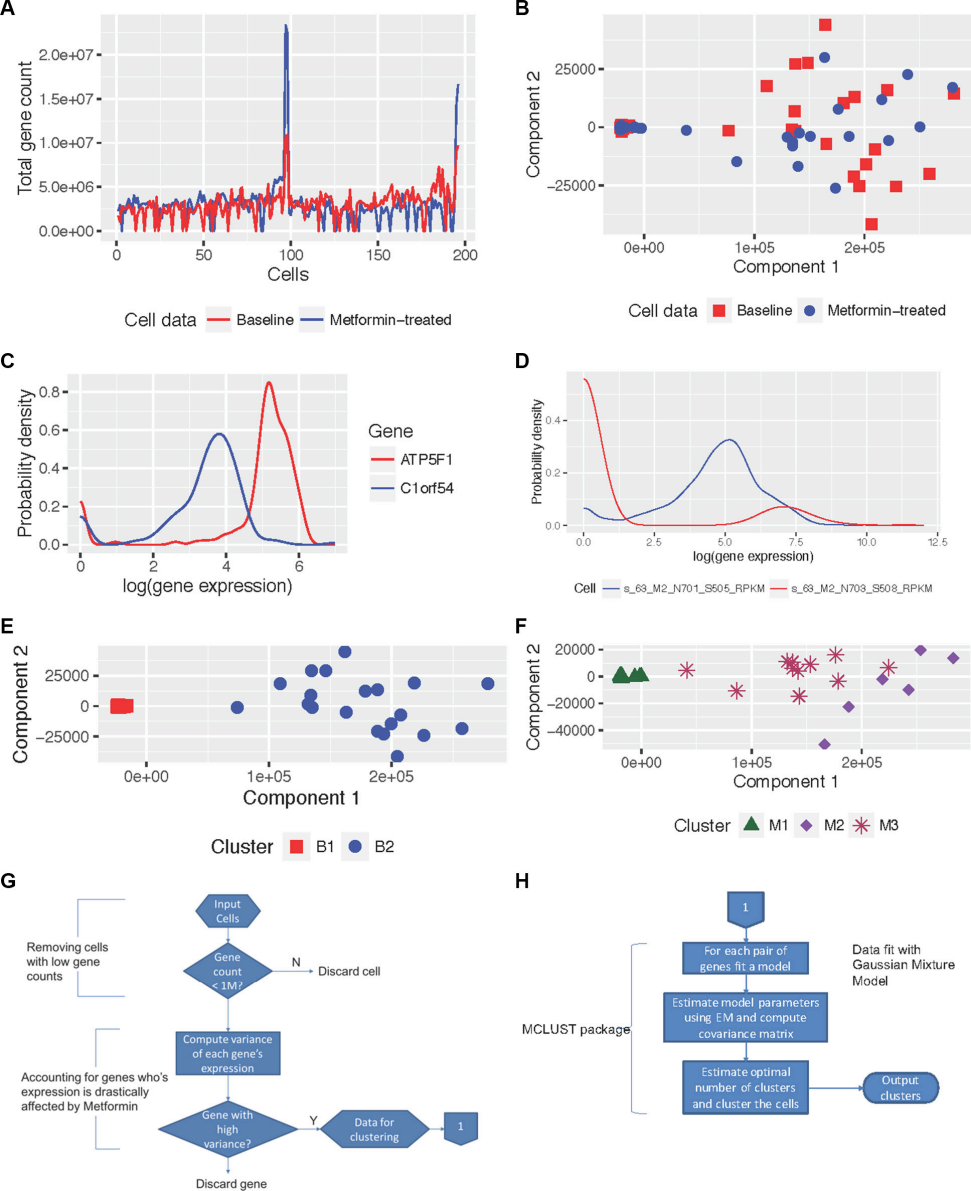
Illustration of data characteristics and results from MiMoSA (**A**) Illustration of high quality in sequencing (gene count > 1 million) in 90% of baseline and metformin-treated cells. (**B**) Baseline and metformin-treated cells projected along the first two principal components derived from principal component analysis (PCA). (**C**) and (**D**) Probability density functions (PDF) of gene expression in genes across cells, and in cells across genes, respectively. These figures show that both PDFs have mixtures, and distributions are also different. (**E**) Baseline cells clustered into two clusters B1 and B2 by MiMoSA and projected using the first two principal components derived from PCA. (**F**) Metformin-treated cells clustered into three clusters M1, M2, and M3 by MiMoSA and projected using the first two principal components derived from PCA. (**G**) and (**H**) Illustrations of stage 1 and stage 2 of MiMoSA.

Cells were treated with 1-mM metformin for 72 hours before RNA was isolated for single-cell sequencing. Duplicate assays were performed for baseline and post-metformin treatment. Therefore, we sequenced 192 cells at baseline and 192 after metformin treatment, referred to subsequently as *baseline cells* and *metformin-treated cells*, respectively. After quality control was performed with MAP-RSeq [14], the initial analysis was conducted on normalized gene expression data. The sequencing data showed that 20% of the genes had a reads-per-kilobase-per-million mapped reads (RPKM) measure greater than 32 (a heuristic for an acceptable gene expression level to indicate that the gene is expressed [14]) in 80% or more of the cells. In the baseline and metformin-treated data sets, 20 and 18 out of 192 cells, respectively, had total gene counts under one million, as shown in Figure 1A, and were excluded from the study. The probability density function (PDF) of gene expression within each cell showed mixtures, as illustrated in Figure 1C. This observation was expected, since not all genes are expected to be uniformly expressed. In addition, the PDF of gene expression also had mixtures, as shown in Figure 1D. We observed that 90% of the information in the PDF was contained in at least two mixtures of the genes. Further, 80% of the genes in both baseline and metformin-treated cells were not expressed in at least 90% of the cells. We chose to study the top fifth percentile of genes (1,170 genes) that were expressed in at least 90% of the cells and were also among those with the highest variance.

### Baseline and metformin-treated cells

To spatially visualize the baseline and metformin-treated cell populations, we combined the two cell populations and projected them onto the first two principal components (which accounted for 97% of the observed variability) from principal component analysis (PCA), as shown in Figure 1B. We observed that in both cell populations, a group of cells was predisposed to a different behavior (right half of the plot) and that metformin-treated cells, in general, exhibited slightly different variability than did baseline cells (spatial separation between baseline and metformin-treated cells).

### Clusters in baseline and metformin-treated cells

For the baseline cells, MiMoSA identified two clusters, B1 and B2, which contained 158 and 20 cells, respectively. A visualization of these clusters along the first two principal components is shown in Figure 1E. We observed that the two clusters behaved similarly, with very little variation in their average expression levels for all genes except for those few that were differentially expressed between the two clusters. This observation indicated that the baseline cells were from a homogenous cell population.

For the metformin-treated cells, MiMoSA identified three clusters, M1, M2, and M3, which contained 160, 6, and 12 cells, respectively. The three clusters are shown projected along the first two principal components in Figure 1F. Clusters M1 and M3 behaved similarly with respect to average gene expression, with slightly higher expression levels in cluster M1. However, cluster M2 was significantly different from clusters M1 and M3 through its spatial separation from M1 and M3, as shown in Figure 1F. Using hierarchical clustering, we were able to obtain the same clusters in metformin-treated cells (Supplementary Section 1).

### Differentially expressed genes

**Figure 2:**
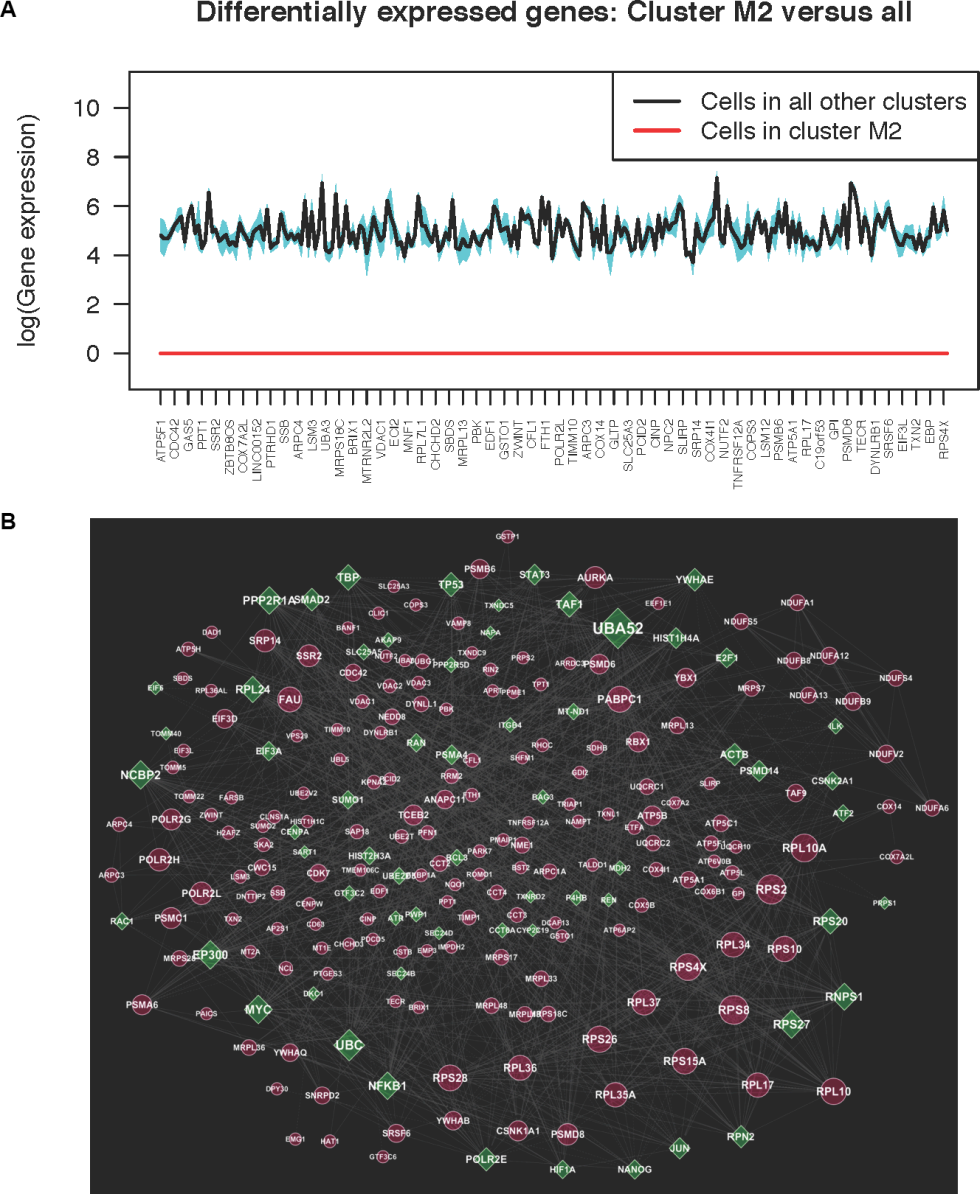
(**A**) The average expression (log scale) of 230 genes (label tics show only a fourth of the 230 genes) that were completely suppressed in cluster M2 in metformin-treated cells, but expressed in all other baseline and metformin clusters. We observe that with two standard deviations around the mean (shaded region), the expressions in clusters except M2 are exhibiting less variance. (**B**) Network analysis of the 230 genes differentially expressed in one of the MDA-MB-231 metformin-treatment clusters compared to all other baseline and metformin-treatment clusters. Red indicates genes from the 230 differentially expressed gene set, and green indicates linker genes obtained from publicly available databases or the literature.

We analyzed all five clusters together to determine specific patterns that might maximize the heterogeneity. We found that 230 genes (Supplementary Table 2) were expressed with RPKM greater than 32 in all clusters of both data sets (B1, B2, M1, and M3) except for cluster M2 in metformin-treated cells, in which these genes were completely suppressed. Figure 2A shows the complete down-regulation of these 230 genes in cluster M2 compared with all other cells in clusters B1, B2, M1, and M3; the width of the shaded region of the plot is set to one standard deviation, and we see that the variation of gene expression for each of the 230 genes in these cells is very small (Figure 2A). To test the statistical significance of the expression values for these 230 genes in M2 in comparison with all other clusters, we performed a Mann-Whitney *U-test* and Kolmogorov-Smirnov test (KS-test), where all expression values of these 230 genes in M2 were compared with their expression values in all other clusters. The *p-value* of this observation for the 230 genes in M2 was 0.00552 (*p-value* of 0.00076 in the KS-test), making it statistically highly significant. Therefore, at the 0.05 significance level, we rejected the null hypothesis and concluded that the expression levels of the 230 genes in M2 and in the other clusters belonged to different populations. No other combination of genes from cluster analysis showed such dramatic changes in gene expression across clusters.

### Pathway-network analysis

We performed network analysis with 230 genes that were observed to be down regulated in one metformin cluster (cluster M2) and upregulated in all other metformin and baseline clusters, as shown in Figure 2B. The 230 gene network was enriched with 15 ribosomal proteins, 8 adenosine triphosphate (ATP) synthases, H+ transporting, mitochondrial complex proteins, 6 cytochrome c oxidases, and 9 nicotinamide adenine dinucleotide (NADH) dehydrogenases. Pathway analysis of the 230 gene network consisted of several enriched metabolic pathways (FDR < 0.005), including the citric acid (TCA) cycle and respiratory electron transport, oxidative phosphorylation, mitochondrial dysfunction, and neurological disease pathways (listed in Supplementary Table 2). The majority of the genes (69%) listed in Supplementary Table 2 were associated with more than two pathways (as shown in Supplementary Table 1) and were supported by multiple literature evidences in the Reactome [15] and KEGG [16] databases. Since, our goal was to identify novel key regulators involved in regulation of metformin treatment in breast cancer, we selected 24 genes for further analysis, genes that are present in the list of significant pathways (bolded and highlighted in Supplementary Table 2), genes that have less experimental evidence showing gene-gene interactions, or genes for which the mechanisms involved transcription regulation are not well understood in the context of metformin treatment. Among these 24 genes, TALDO1, PRPS2, PTGES3, RRM2, DAD1, PAICS, PARK7, RPL36AL, SRP14, SSR2, CDC42 have been shown to be down regulated in the presence of metformin in a rat hepatoma line and breast cancer cells [17, 18].

In addition, *CDC42*, has also been shown to be down-regulated in breast cancer patients treated with metformin. However, the exact mechanism involved in regulation of CDC42 in response to metformin treatment is not yet known, thereby making it a candidate for our functional studies.

### Metformin inhibits expression of CDC42

CDC42 is a ubiquitously expressed small guanosine triphosphatase (GTPase). It is known to regulate the dynamic organization of the cytoskeleton and membrane trafficking for physiologic processes, such as cell proliferation, mortality, polarity, cytokinesis, and cell growth. Deregulation of CDC42 can alter the normal function of cells and has been associated with cancer development and tumor metastasis [19]. Metformin has been shown to inhibit cell migration and metastasis, possibly through its inhibition of EMT and cancer stem cells [20–22]. TNBC is exquisitely sensitive to metformin treatment. Mechanistically, metformin dampens TGF-β signaling, resulting in abrogation of EMT. Further, metformin can also reduce migration and invasion of mesenchymal stem-like cells [23]. It is not known whether or how CDC42 might be involved in metformin-induced cellular phenotypes, especially the inhibition of cell migration, so we performed functional studies to pursue the possible mechanism(s) involved in CDC42's contribution to metformin effect in these cells.

**Figure 3:**
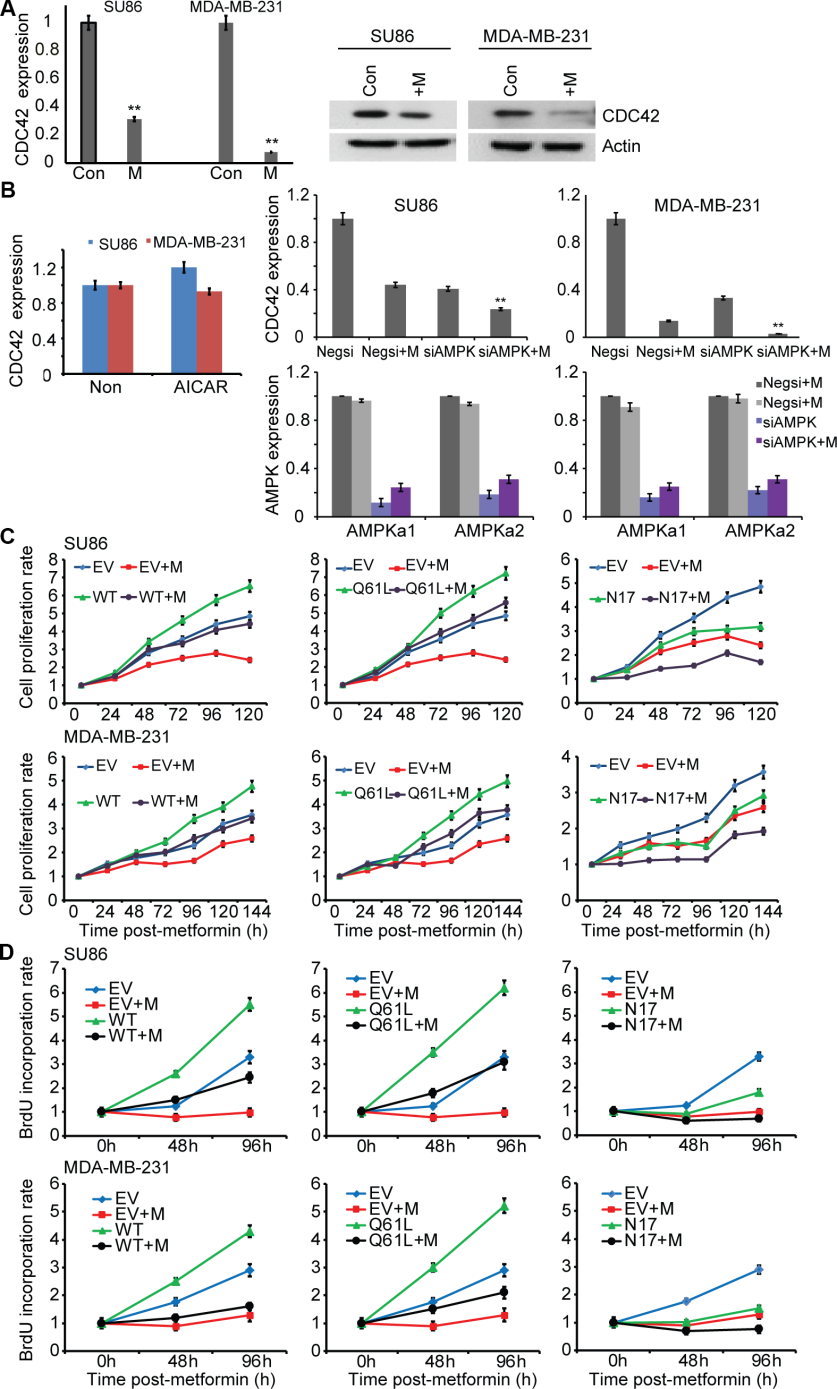
Metformin inhibits expression of CDC42 (**A**) The effects of metformin treatment on the endogenous mRNA expression levels of CDC42 in SU86 pancreatic cancer cells and MDA-MB-231 breast cancer cells were determined by qRT-PCR and western blotting. SU86 and MDA-MB-231 cells were treated with 5mM and 1mM metformin, respectively, and 30 μg lysate were analyzed by western blotting using antibodies against CDC42 and actin, a loading control. Target gene expression was normalized to the reference gene, GAPDH, and represented as mean fold change relative to vehicle treatment. Error bar represents the mean fold changes ± SEM of 3 biological triplicates. (**B**) Effects of AMPK on metformin-induced decrease in CDC42 mRNA expression in SU86 and MDA-MB-231. Cells were treated with AICAR or were co-transfected with AMPKα1 and AMPKα2 siRNAs followed by incubation with or without metformin (5 mM for SU86 and 1 mM for MDA-MB-231) for 48 hours. Quantification of CDC42 mRNA levels and AMPK knockdown efficiency is shown. (**C**) CDC42 is involved in metformin-mediated growth inhibition. SU86 and MDA-MB-231 cells were transfected with empty vector or CDC42 WT and mutant constructs, and then treated with metformin at different concentrations for 24, 48, 72, 96, 120, and 144 hours. Cell proliferation was measured by MTS assay. The results are represented as the mean ± SD of three independent experiments. CDC42 knockdown efficiency was determined by qRT-PCR and western blot. *p < 0.05; **p < 0.01. (**D**) BrdU incorporation assays were performed at 48 and 96 hours after metformin treatment in SU86 and MDA-MB-231 cells transfected with empty vector or CDC42 WT and mutant constructs. Values are shown as mean ± s.d.

As a first step, we confirmed metformin's effect on the down regulation of CDC42. Specifically, we determined CDC42 expression levels in human MDA-MB-231 triple-negative breast cancer cells before and after metformin exposure and observed a striking down regulation of CDC42 expression in the presence of metformin (Figure 3A). To further confirm that this phenomenon was not cell-type-specific, we also used SU86, a pancreatic cancer cell line, and repeated the experiment (Figure 3A, left panel). We observed a significant decrease in CDC42 mRNA and protein levels in response to metformin (Figure 3A, right panel). To determine whether metformin down regulation of CDC42 at the transcriptional level depended on AMPK, SU86 and MDA-MB-231 cells were treated with 5-aminoimidazole-4-carboxyamide ribonucleoside (AICAR), an activator of AMPK (Figure 3B). Treatment with AICAR did not significantly change CDC42 expression. We also performed knockdown of AMPKα1 and AMPKα2 in those two cell lines. Knocking down AMPK did not reverse metformin-induced down regulation of CDC42 expression. In fact, down regulation of AMPK also resulted in decreased CDC42 expression (Figure 3B). Metformin treatment can activate AMPK. If AMPK activation is required for metformin-induced down regulation of CDC42, we would have expected a reversal of CDC42 transcription level after the down regulation of AMPK. Therefore, taken together, these experiments suggested that metformin-induced down regulation of CDC42 expression levels was independent of AMPK activation, even though AMPK itself can regulate CDC42 expression.

### Knockdown of CDC42 and treatment with metformin reduces breast cancer cell proliferation

We next investigated the effect of CDC42 on metformin response and cancer cell proliferation using both MTS assay and BrdU labeling [24]. It was shown previously that inhibition of CDC42 can decrease cancer cell proliferation [25] and that metformin can inhibit cell proliferation. We found that overexpression of wild type (WT) CDC42 can reverse the decrease in cell proliferation observed after metformin treatment when compared with empty vector transfected cells treated with metformin using both MTS assay and BrdU labeling assay (Figure 3C–3D, left panel). Overexpression of a constitutively active CDC42 (Q61L) construct showed a similar effect on cell proliferation in the presence of metformin (Figure 3C–3D, middle panel). However, overexpression of a dominant negative CDC42 (N17) construct did not reverse the metformin-induced inhibition of cell proliferation (Figure 3C–3D, right panel). These results indicate that metformin-mediated growth inhibition involves CDC42.

### Metformin inhibits migration and invasion of cancer cells by inhibiting CDC42

**Figure 4:**
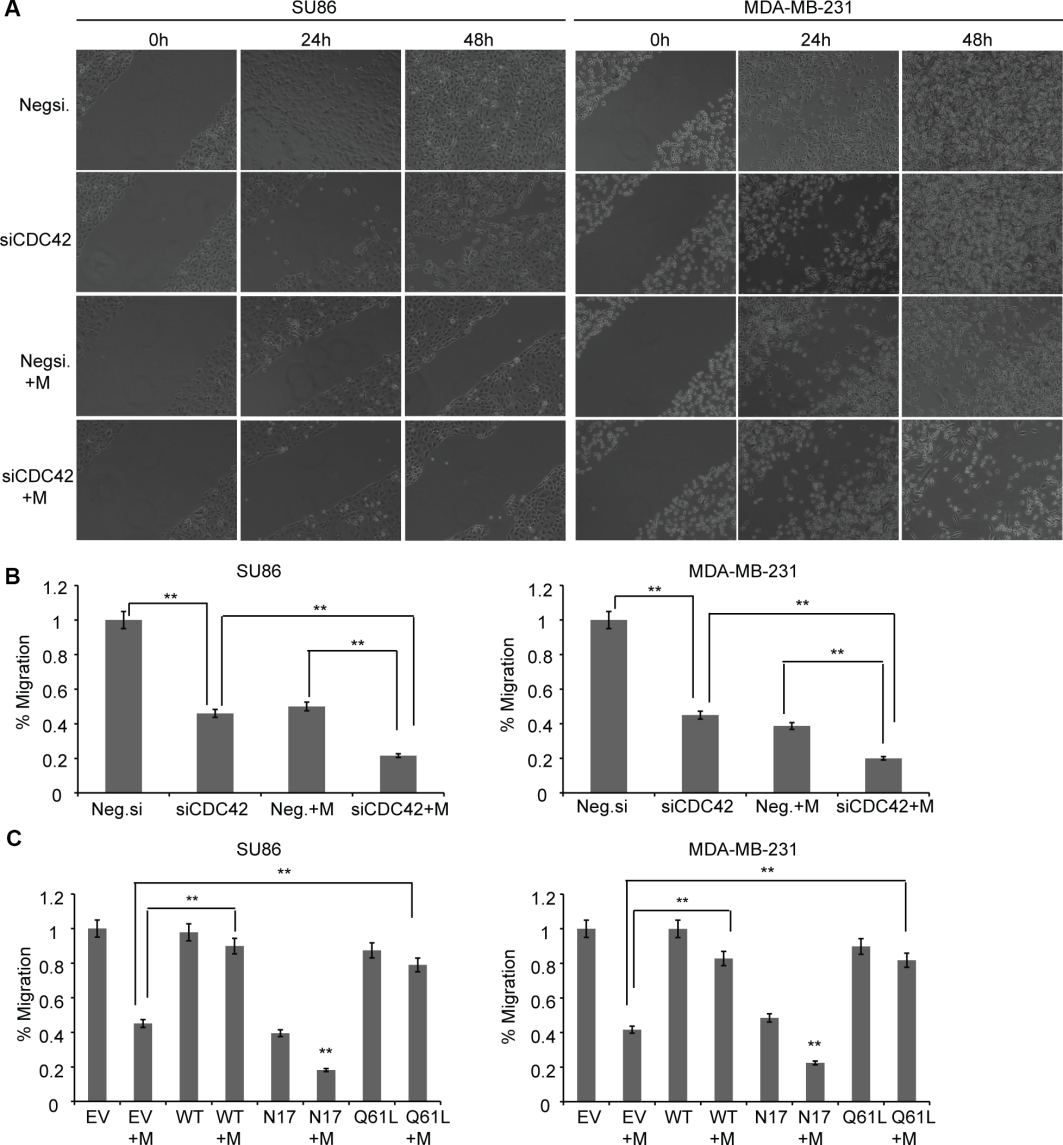
Metformin regulates cancer cell migration and invasion by down regulation of CDC42 (**A**) Metformin regulates cancer cell migration by reducing the expression of CDC42. SU86 and MDA-MB-231 cells were transfected with Negsi (negative control siRNA) or siCDC42 (siRNA specific for CDC42). Equal numbers of cells from Negsi and siCDC42 groups were seeded into 12-well plates for wound healing assay. Metformin (1–5 mM) was added during the migration for 48 hours. Original magnification: ×10. (**B**) SU86 and MDA-MB-231 cells were transfected with Negsi (negative control siRNA) or siCDC42 (siRNA specific for CDC42), and then treated with or without metformin. Cell invasion was analyzed 24 h post transfection using Transwell invasion assay. Group *t*-tests were performed to compare each data point with the control (siCon). Error bars are mean ± SD (*n* = 3). **p < 0.01. (**C**) SU86 and MDA-MB-231 cells were transfected with empty vector (EV), wild type CDC42 expressing plasmid (WT), N17 (dominant negative), or Q61L (constitutively active), and then treated with or without metformin. The Transwell invasion assay was performed as described above. Error bars are mean ± SD. (*n* = 3). **p < 0.01.

Recent studies have demonstrated that metformin can block invasion and metastasis for several types of cancers [26–30]. However, the underlying mechanism is not well understood. CDC42 plays a key role in regulating actin dynamics [31–33] and promotes the transendothelial migration of cancer cells [34]. We set out to determine whether CDC42 might be an important factor in mediating the effects of metformin on cell migration. Specifically, we transfected CDC42 siRNA into SU86 and MDA-MB-231 cells and found that CDC42 knockdown decreased the migration of both SU86 and MDA-MB-231 cells using a wound-healing assay (Figure 4A). When a matrigel invasion assay was used, knocking down of CDC42 also significantly inhibited the invasion of SU86 and MDA-MB-231 cells (Figure 4B). Treatment of CDC42 knocked-down cells with metformin showed a synergistic effect on the inhibition of cell migration, indicating that CDC42 might contribute to metformin-dependent inhibition of cell migration. This possibility was confirmed when cells were transfected with the wild type (WT), constitutively active (Q61L), and dominant negative CDC42 (N17) plasmids (Figure 4C). The inhibitory effect of metformin on migration was partially reversed in cells transfected with WT or constitutively active CDC42 (Q61L), whereas metformin consistently elicited a greater inhibition of migration in cells transfected with dominant negative CDC42 (N17). These results suggest that metformin suppresses cancer cell migration through a CDC42-mediated signaling pathway.

**Figure 5:**
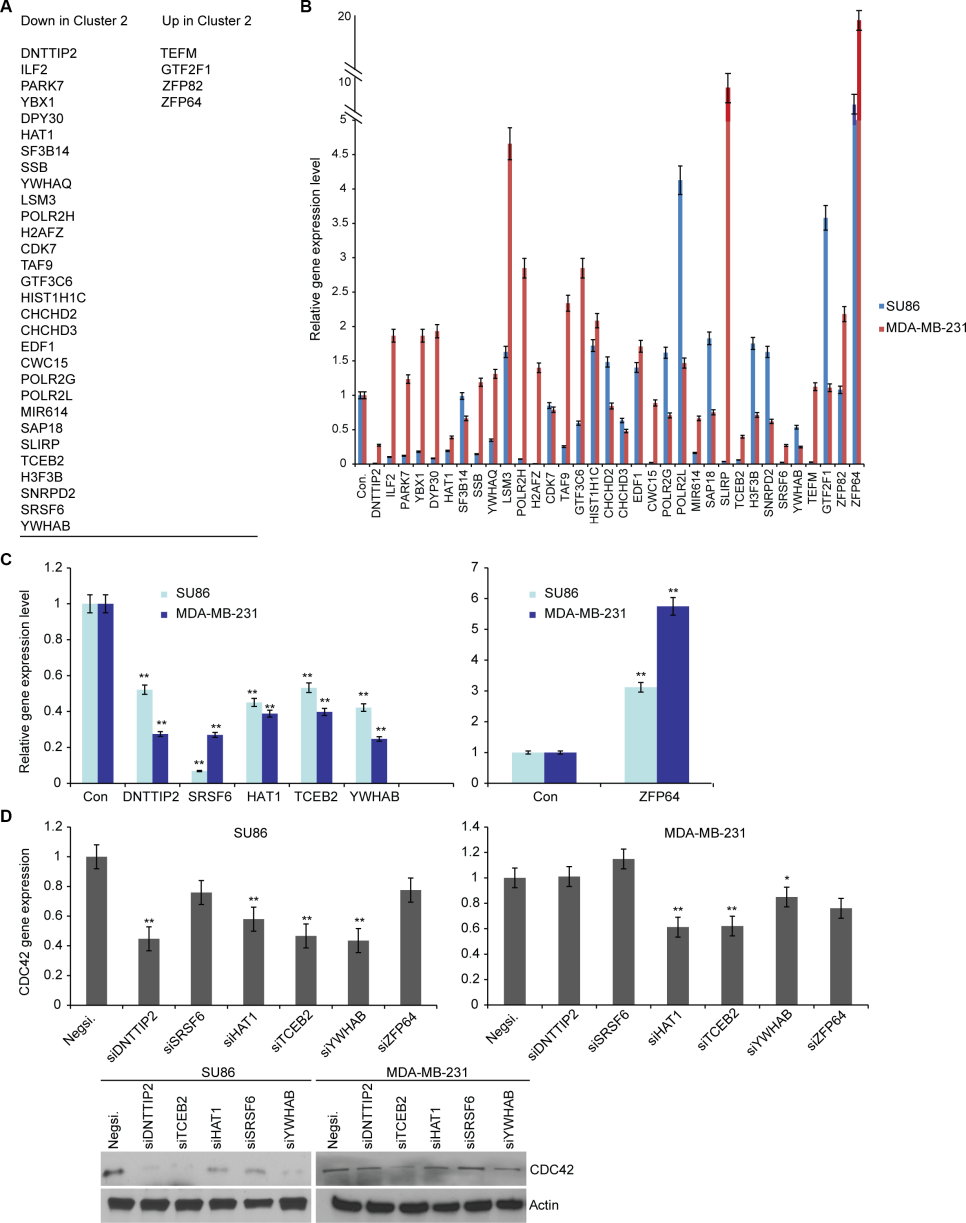
Metformin regulates CDC42 expression by regulating transcription regulatory genes (**A**) Candidate transcription regulatory genes from the scRNA-seqanalysis that differentiate cluster M2. Candidate transcription regulatory genes were chosen from the list of genes expressed in clusters M1 and M3 but not expressed in cluster M2, and genes not expressed in clusters M1 and M3 but expressed in cluster M2. (**B**) Metformin-induced expression changes for the transcription regulatory genes. SU86 and MDA-MB-231 cells were treated for 48 hours with 1–5 mM metformin prior to RT-qPCR analysis of endogenous mRNA expression levels of the indicated genes. Gene expression was normalized to the reference gene GAPDH and presented as mean fold change relative to vehicle treatment. Error bars represent mean fold changes ± SEM of biological triplicates. One representative experiment is shown. **p < 0.01. (**C**) Candidate transcription regulatory genes that are consistently deregulated in both SU86 and MDA-MB-231 cells by metformin. (**D**) siRNA screening of candidate transcription regulatory genes for the regulation of CDC42. Quantification of endogenous mRNA levels of CDC42 in SU86 and MDA-MB-231 cells was measured by RT-qPCR 48 hours after transfection with negative or specific siRNA. Gene expression was normalized to reference gene GAPDH and presented as mean fold change relative to transfection with Negative siRNA ± SEM of biological triplicates of a representative experiment. Protein levels of CDC42 and load control actin were assessed by western blot 48 hours after transfection of cells with specific siRNA. **p < 0.01.

To determine how metformin affects *CDC42* gene transcription, we reviewed the list of 230 genes that are differentially expressed in cluster M2 cells treated with metformin. We chose all of those genes that are known to be involved in transcription regulation. We used a literature search to identify 34 candidate genes that play a role in transcription regulation (Figure 5A). Treatment with metformin led to significant changes in the expression of several of those candidate genes in SU86 and MDA-MB-231 cells (Figure 5B). We then identified five candidate transcription regulators that were consistently down regulated and one gene, *ZFP64*, that was significantly up regulated in cell lines after metformin treatment (Figure 5C). To determine whether these genes might directly affect *CDC42* expression, SU86 and MDA-MB-231 cells were transiently transfected with siRNAs targeting each of the six candidate genes. Knockdown of deoxynucleotidyltransferase terminal-interacting protein 2 (DNTTIP2), histone acetyltransferase 1 (HAT1), transcription elongation factor B polypeptide 2 (TCEB2), and 14-3-3 protein beta/alpha (YWHAB) decreased *CDC42* gene transcription in SU86 cells, whereas knockdown of HAT1, TCEB2, and YWHAB decreased *CDC42* gene transcription in MDA-MB-231 cells (Figure 5D, upper panel); those results were also confirmed by western blot analysis (Figure 5D, lower panel). These results suggest that metformin's effect on *CDC42* transcription might be mediated through the regulation of several transcription regulators, including DNTTIP2, HAT1, TCEB2, and YWHAB.

## DISCUSSION

Single-cell RNA sequencing has promised to uncover transcriptome variability in subpopulations of cells (clusters) that otherwise would have been missed with bulk sequencing methods. However, with limited samples for analysis and the presence of noise, novel methods are needed to analyze single-cell data; further, in most cases, the methods need to be tailored to specific data characteristics.

In this study, we applied a data-driven unsupervised analysis approach, in which the clustering problem was driven by the distribution of gene expression profiles of the cells to identify candidate genes for functional analysis [9]. That helped identify a unique cluster of only 6 cells that showed the most dramatic effect in the expression of 230 genes that were down regulated after exposure to metformin. Focusing on the subset of the 230 genes that was related to known metformin and breast cancer pathways with sufficient statistical significance, we found that 24 genes were less studied (present only in one one pathway with FDR < 0.005) Included among these 24 genes was *CDC42*, down-regulated in breast cancer treated with metformin. Functional studies revealed this gene to be involved in metformin inhibition of triple-negative breast cancer cell migration. This result combines a novel method of unsupervised learning analysis that allowed us to decide on choosing *CDC42* as a candidate for functional studies. An extensive functional evaluation of the method's findings provides novel insights into the role of CDC42 in metformin response.

In our study, we used the model-based unsupervised learning method to solve a machine-learning problem defined as follows: cells are the data samples, and genes are the features; gene-expression behavior in samples will be used to infer cell types. One common observation in all extant work on scRNA-seq analysis is the high-dimensional nature of the problem. In our study, by combining two assays for each dataset, we have a total of 192 cells (data samples) in our analysis to infer cell heterogeneity using 23,398 genes (features). Thus, the number of data samples is at least two orders of magnitude smaller than the number of features that are used to differentiate the samples, making single-cell analysis a high-dimensional problem. Further, by excluding genes that either are not expressed or show similar expression across all samples, earlier work has found that only a small number of genes may contribute to cell heterogeneity [35–37].

To reduce the number of genes used to infer cell heterogeneity, we chose the top fifth percentile of the expressed genes with highest variance. This reduced the ratio of the number of genes to the number of cells by at least one order of magnitude while still capturing 95% of the expression variability in two principal components. We found no significant difference in either the raw counts or the natural log-transformed data in terms of results. However, we decided to present our results using raw RPKM measures, for two reasons. First, the idea behind using the two principal components to project the results was that genes with maximum variability drive the separation of the points in lower-dimensional space. Second, the first two principal components accounted for 96.5% and 0.5% of the observed variability, indicating that only a few genes were driving variability in the dataset. Hence, using the raw RPKM measure would offer a larger divergence of the cells’ PDF than a log transformation of the RPKM measures would.

The top 5% high-variance genes all have over one million sequence reads. As shown in Figure 1A, we found that a fraction of cells had sequencing depths of under one million reads and excluded them from our study. While there is significant interest in the single-cell research community in determining the optimum sequencing depth per cell for analyses, there is still no standard available to decide the optimal sequencing coverage. Several studies have shown that low sequencing depth also allows for the identification of subpopulations of cells [38–40]. However, there is also evidence that one million reads suffices to study single cells and that higher sequencing depth is preferred when studying subtle differences among cells [41, 42]. With these findings, we justified a higher sequencing depth because we wanted to seek subtle variation that metformin introduces in cells. Hence in this work, we decided that a sequencing depth of one million reads was a reasonable threshold for this work.

In this work, we assumed that without loss in generality, the distributions of clusters were finite Gaussian mixture models with exponential distributions. This choice of clustering method augurs well for our observation that gene expressions in our data for a large population of cells (∼100 cells) had mixtures in their distributions and provided the best model fits for Gaussian mixture models (GMM) with varying volume and equal shape (See Supplementary Figure 1). Using the estimated means and variance of the mixture model, we generated 10,000 random samples. To test whether the PDF of the generated samples was statistically close to the PDF from the data, we performed a Mann-Whitney *U-test*. For illustration, we arbitrarily chose a cell, and for this cell the *p-value* from the test was 0.7406. At a significance level of 0.05, we failed to reject the null hypothesis that the gene expression of the cell chosen and the generated expression levels from the estimated mixture model were drawn from identical populations; the result can also be visually verified by comparing the PDFs in Supplementary Figure 1. Thus, we made this work truly data-driven by allowing the clustering to be determined by distributions in the data instead of by distances or densities. The use of distances or densities might require stricter assumptions with regard to data characteristics (e.g., normality) and will not guarantee consistent answers if the feature space and its associated variability are large.

By comparing the clusters of cells from MiMoSA, we identified 230 genes that were significantly down regulated in one cluster (M2) of metformin-treated cells but were uniformly well expressed in all other clusters (*p-value* of 0.00552), as shown in Figure 2A. This finding raised the question of whether these 230 genes might be markers for metformin sensitivity in triple-negative breast cancer cells. Metformin has been used to treat type 2 diabetes for decades, but its antitumor effects were not recognized until recently. Regulation of the AMPK pathway is thought to be a major mechanism of metformin's antitumor activity [6]. Metformin-induced inhibition of oxidative phosphorylation leads to elevated levels of cellular AMP, which activates the energy sensor AMPK [43]. Activated AMPK phosphorylates the tuberous sclerosis complex (an inhibitor of mammalian target of rapamycin (mTOR)), leading to the up regulation of its activity and subsequent mTOR inhibition and tumor suppression [44]. Despite increasing knowledge about metformin's anticancer mechanisms, the exact mechanisms responsible for these actions remain unclear.

In follow-up functional analyses, we selected one of the 230 genes that we found to be down regulated by M2: CDC42, a major player in actin dynamics that is also down regulated in breast cancer patients treated with metformin. Metformin has been shown to inhibit cell migration and tumor metastasis [26–30]. One mechanism involves metformin inhibition of cancer stem cells [45, 46]. We therefore also examined a number of mesenchymal markers described previously [47], including SOX2, CD44, CD24, CD133, ALDH, OCT-4, FN1, SNAI2, VIM, FOXC2, MMP2, MMP3, CDH1, and DSP, at the mRNA level in all clusters. All of these genes except *VIM* and *CD44* were suppressed in all clusters (baseline and metformin-treated cells); VIM and CD44 were equally expressed in all three clusters without any differential behavior among the clusters, either at baseline or metformin-treated cells. Our results suggested additional mechanisms by which metformin might have an effect on cell proliferation and cell migration, mechanisms that are not entirely dependent on AMPK activation. AMPK activation was not required for metformin-induced down regulation of CDC42 expression (Figure 3B). However, AMPK could up-regulate CDC42 expression, since knockdown of AMPKα1 and AMPKα2 dramatically reduced CDC42 level (Figure 3B). We did not observe any increase or other change in CDC42 level in the presence of AICAR, a known AMPK activator, probably because of the very high baseline level of CDC42. These observations together, the non-AMPK-dependent effect on the down regulation of CDC42 by metformin was a major determinant of CDC42's effect on cell proliferation and cell migration. The reason was that metformin's ability to inhibit cell proliferation and cell migration was increased when CDC42 expression was down regulated (Figure 3C–3D, Figure 4). Furthermore, we found that the regulation of CDC42 expression by metformin occurred, at least in part, through the regulation of a series of genes involved in transcription regulation, including DNTTIP2, TCEB2, and YWHAB (Figure 5). DNTTIP2 is involved in chromatin remodeling and gene transcription [48]. HAT1 is a type B histone acetyltransferase involved in the rapid acetylation of newly synthesized histones. Evidence suggests that HAT1 moderates the nuclear factor κB (NF-κB) response by regulating the transcription factor promyelocytic leukemia zinc finger (PLZF) [49]. TCEB2, a subunit of the transcription factor B (SIII) complex, is known to activate elongation by RNA polymerase II [50], and YWHAB acts as a coactivator for several transcription factors, including Peroxisome proliferator-activated receptor γ2 (PPARγ2) [51].

Notably, several studies demonstrate that metformin is beneficial for neurodegenerative disease such as Alzheimer′s disease and Huntington's disease [52, 53]. Recently, several studies also reported the neuroprotective effect of metformin in Parkinson's disease model [54, 55]. Energy metabolism disturbance and mitochondrial dysfunction have been implicated in the pathogenesis of Parkinson's disease [56]. Interestingly, our pathway analysis of the 230 genes revealed pathways related to Parkinson's disease, Huntington's disease, and Alzheimer's disease with over 20 genes in each pathway (Supplementary Table 1). These pathways are deregulated in cluster 2 post metformin treatment, suggesting metformin may play a role in neurodegenerative disease. However, further studies are needed in order to determine whether metformin's effect on neurodegenerative disease contributes to the therapeutic effect of metformin in the treatment and prevention of TNBC.

In summary, MiMoSA identified cluster M2, which contained only six cells that showed significantly differential gene expression with plausible biological significance after exposure to metformin. In all engineering and applied-statistics practices, such clusters are generally discarded as outliers. Thus, it is possible that this is the kind of subtle-yet-important cell behavior that we can pursue using a combination of single-cell sequencing technology and data-driven unsupervised learning methods. Our results also provide a series of metformin exposure genes. This 230 gene signature needs to be further validated in other cell lines and clinical samples. We understand that gene expression has tissue specificity; therefore, it is important to determine the effect of metformin on these genes in different cells or tissue types. Our study also has provided novel insights into the metformin effect on CDC42 through the regulation of a series of transcription factors. Future studies should identify how these transcription regulators influence CDC42 gene expression. The relationship could also be tested further using clinical samples and by determining metformin outcomes.

## MATERIALS AND METHODS

### Cell lines

Human triple-negative breast cancer cell line MDA-MB-231 and pancreatic cancer cell line SU86 were obtained from the American Type Culture Collection (Manassas, VA). MDA-MB-231 cells were cultured in L-15 medium containing 10% fetal bovine serum (FBS). SU86 cells were cultured in Roswell Park Memorial Institute (RPMI) 1640 medium containing 10% FBS.

### Experimental setup and data generation

The primary dataset included 384 scRNA-seq samples from MDA-MB-231 cells, half of which had been exposed to metformin. Specifically, MDA-MB-231 cells were plated into 6-well plates (6 × 10^4^ cells per well) and incubated for 24 hours; half were then exposed to 1 mmol/L metformin (Sigma-Aldrich, St. Louis, MO). Fresh medium and drug were replaced daily; after 5 days, cells were resuspended and single cells immediately captured with the Fluidigm C1 system. Two independent MDA-MB-231 batches were used, and 96 untreated cells (control) and 96 metformin-treated cells were captured from each. Cells from the two independent batches were captured on two different C1 machines in an orthogonal design. Sequencing libraries were prepared with the standard Fluidigm protocol based on SMARTer chemistry and Illumina Nextera XT. RNA sequencing of 100-bp paired-end reads was done on an Illumina HiSeq, with an average of 4.9 million reads/cell.

### Data preprocessing

Raw Fastq files for 384 RNA-seq single cells were obtained and processed through the MAP-RSeq workflow [14] to obtain binary alignment map (BAM) files, summary alignment statistics, and quality control visualizations of mapped reads for each single cell. Paired-end RNA-seq reads were aligned using TopHat [57] to the human genome build NCBI 37.1, which corresponds to the hg19 human genome assembly from the University of California, Santa Cruz (UCSC; [58]). Total reads, mapped reads, unmapped reads, and junction reads were obtained for each single cell to identify samples with no cells or low reads. We removed 19 and 15 samples with a total count of < 1 million reads from further analysis in baseline and metformin-treated cells, respectively, and the average total reads in baseline and metformin-treated cells were 3.10 million and 3.02 million, respectively. The gene-expression count data for single cells was obtained using HTSeq software [59].

### Network and pathway analysis

Network analysis was performed using Cytoscape [60], NetworkAnalyzer [61], and the Reactome database [62] functional interaction (FI) feature. Linker genes were included in the network analysis. Through the Reactome database, and based on information obtained from the literature or on data evidence, these linker genes were connected in the network with the gene set we were investigating. Network parameters, such as number of edges for each node, neighborhood connectivity, and degree of connections for each node, were obtained to determine the size of the node in the network. The size of the node in the network indicates the degree of connection with other genes. Hence, the size of the nodes in the network indicates the gene connectivity. The pathway enrichment analysis method in Cytoscape was applied to identify pathways enriched in the network. Gene annotations were obtained using the UCSC refFlat file.

### Mixture model-based single-cell analysis (MiMoSA)

As the best fit for the mixtures in our data was Gaussian mixture models (GMM), we used MiMoSA to cluster the cells so that the method makes use of the GMM. The MiMoSA workflow uses a model-based clustering algorithm tailored for data that exhibit mixed Gaussian distributions in the PDFs [9]. The workflow is divided into two stages, as illustrated in Figure 1G and 1H.

Stage 1 (Figure 1G): Inputs to this step are the baseline and metformin-treated RPKM measures from the MAP-RSeq workflow. [Step i] *Remove cells with low gene counts*: We remove cells from the analysis if they have a total gene count under one million. This allows us to study cells with high sequencing depth and quality. [Step ii] *Eliminate genes with low variance*: The goal here is to understand the mechanism of metformin in breast cancer cells. Thus, to observe metformin's action, we wanted to capture the maximum variability in gene expression introduced by metformin in comparison with baseline cells. We observed that 80% of the genes were found with low expression (with RPKM less than 32), consistent with the existing literature, and hence it made sense to remove these genes from the analysis. In this work, we chose genes that were in the top fifth percentile of variance observed across the whole genome. *This reduced the number of genes we used for clustering cells from 23,398 to 1,170*. We output the cells with the reduced set of genes to Stage 2 of MiMoSA.

Stage 2 (Figure 1H): All computations in stage 2 are performed with an existing R package called MCLUST [63]. The MCLUST package performs model-based clustering that makes use of the Gaussian mixture models, and uses an expectation-maximization algorithm to learn the model parameters. MCLUST has been widely used for applied mathematics and engineering applications. The optimal number of clusters is chosen based on the Bayesian information criteria (BIC) plot: it is the number at which the scree plot for BIC against the increasing number of clusters starts to see an asymptotic convergence. MCLUST outputs a data structure that contains the cluster label for each data sample (cell), among other clustering parameters.

### Quantitative reverse-transcription PCR (qRT-PCR)

Total RNA was isolated from cultured cells with the QIAGEN RNeasy kit (QIAGEN Inc., Valencia, CA, USA), followed by qRT-PCR performed with the one-step Brilliant SYBR Green qRT-PCR master mix kit (Stratagene, La Jolla, CA, USA). Specifically, primers purchased from QIAGEN were used to perform qRT-PCR with the Stratagene Mx3005P™ real-time PCR detection system (Stratagene). All experiments were performed in triplicate with GAPDH as an internal control. Reverse-transcribed Universal Human Reference RNA (Stratagene) was used to generate a standard curve. Control reactions lacked the RNA template.

### Western blot analysis

MDA-MB-231 cells were lysed with the CelLytic M Cell Lysis buffer (Sigma-Aldrich, St. Louis, MO, USA). Cell lysate was subjected to electrophoresis on 10% sodium dodecyl sulfate polyacrylamide gel electrophoresis (SDS-PAGE) gels, followed by transfer to polyvinylidene fluoride membranes. The polyvinylidene fluoride membranes were blocked with 5% nonfat milk at room temperature for 1 h and then probed with primary antibodies against CDC42 at a 1:1,000 dilution (BD Biosciences, San Jose, CA, USA) and β-actin at a 1:1,000 dilution (Sigma-Aldrich, St. Louis, MO, USA). Protein bands were visualized using enhanced chemiluminescence (Thermo Scientific, Rockford, IL, USA).

### Transient transfection and RNA interference

Specific siGENOME siRNA SMARTpool^®^ reagents against a given gene as well as a negative control, siGENOME Non-Targeting siRNA, were purchased from Dharmacon Inc. (Lafayette, CO, USA). A second CDC42 siRNA was purchased from QIAGEN (Valencia, CA, USA). MDA-MB-231 and SU86 cell lines were used to perform the siRNA knockdown studies. The Lipofectamine RNAiMAX transfection reagent (Invitrogen, Carlsbad, CA, USA) was used for siRNA reverse or forward transfection. Specifically, cells were seeded into 96-well plates or 6-well plates and mixed with siRNA-complex consisting of 20–50 nmol of specific siGENOME siRNA SMARTpool or nontargeting negative control (Dharmacon) and Lipofectamine RNAiMAX transfection reagent.

The CDC42 plasmids that included WT, N17 (dominant negative), or Q61L (constitutively active) constructs were gifts from Dr. Daniel Billadeau, Mayo Clinic Rochester. Plasmids were transfected with Lipofectamine 2000 (Invitrogen, Carlsbad, CA, USA).

### MTS cytotoxicity and cell proliferation assay

Metformin was purchased from Sigma-Aldrich (Milwaukee, WI, USA). Drugs were dissolved in phosphate-buffered saline (PBS), and aliquots of stock solutions were frozen at −80°C. Cytotoxicity assays were performed in triplicate at each drug concentration. Specifically, cells were seeded onto 96-well plates and mixed with siRNA-complex consisting of 20–50 nmol of specific siGENOME siRNA SMARTpool or nontargeting negative control (Dharmacon) and the Lipofectamine RNAiMAX transfection reagent [64]. After 24 hours, cells were treated with 10 μL of metformin at final concentrations of 0, 0.39, 0.78, 1.56, 3.125, 6.25, 12.5, 25, 50, and 100 mmol/L. After incubation for an additional 72 hours, 20 μL of CellTiter 96^®^ Aqueous Non-Radioactive Cell Proliferation Assay solution (Promega Corporation, Madison, WI, USA) was added to each well. Plates were read in a Safire2 plate reader (Tecan AG, Switzerland) [65].

The MTS assay was used to determine whether metformin altered cell proliferation. Cells were transfected with specific siRNA or plasmids for 24 h; 10^4^ cells were plated in triplicate in 96-well plates and then treated with metformin or vehicle control (dimethyl sulfoxide (DMSO)). MTS assays were performed at 0, 24, 48, 72, 96, 120, and 144 hours after metformin treatment.

Cell proliferation was also measured using the BrdU Cell Proliferation Assay kit (Cell Signaling, Danvers, MA), following the manufacturer's instructions. Specifically, 5000 cells per well were plated on a 96-well plate in triplicate for each of three independent experiments. SU86 and MDA-MB-231 cells were transfected with CDC42 plasmids, and then treated with 1–5 mM metformin for 24, 48, and 96 hours. Cell proliferation was assayed relative to day 0 using the BrdU Cell Proliferation Assay Kit after incubation with bromodeoxyuridine (5-bromo-2-deoxyuridine, BrdU) for 2 h. Cells were then fixed with a fixing solution for 30 min and subjected to immunostaining with a monoclonal anti-BrdU antibody and an HRP-conjugated secondary antibody. After adding substrate of theTMB (tetramethyl-benzidine) peroxidase to the cells, the cells were incubated for 30 min in the dark and the stop solution was added to terminate the reaction. Absorbance was read at 450 nm using in a Safire2 microplate reader (Tecan AG, Switzerland).

### Wound-healing assay

SU86 and MDA-MB-231 cells were seeded in a 6-well plate and grown to confluent monolayers. Next, cells were cultured in medium without FBS for 12 h. A 200-μL pipette tip was drawn across the center of the well to produce a wound area and washed twice with serum-free medium to remove detached cells. Cells were then treated with a medium containing different concentrations of metformin (1 or 10 mM) and 1% FBS. (1% FBS permits cell survival but not cell proliferation.) Subsequently, images of the wound-healing process were photographed digitally (×10) at 0, 24, and 48 h. Each value was an average value derived from three randomly selected fields.

### Cell invasion assay using staining

An invasion assay was performed using 24-well Transwell units with 8-μm-pore-size polycarbonate inserts. Cells were cultured in serum-free medium for 24 h. The polycarbonate membranes were cultured at 37°C for 1 h. A cell suspension (100 μL of 1 × 10^5^ cells/mL in serum-free medium) containing metformin or vehicle was added into the inner cup of a 24-well Transwell chamber, which had been coated with 50 μL of Matrigel™ (BD Biosciences, Franklin Lakes, NJ, USA). 650 μL of culture medium containing 10% FBS was added into the lower compartment as a chemoattractant. After incubation for 24 h, non-invading cells were removed from the upper surface by gentle rubbing with a cotton-tipped swab. Cells that had migrated through the Matrigel and the 8-μm-pore membrane were fixed and stained with 0.1% crystal violet. The lower filter was used to count cell numbers in five random fields under the microscope. Each experiment was performed in triplicate.

### Statistical analysis

All data were presented as mean ± SD of at least three independent experiments. Statistical analysis was performed using SPSS22.0 and Prism 5 (GraphPad Software Inc., San Diego, CA, USA). Single-factor analysis of the variance test was used for comparisons among multiple groups, and a *t-test* was used for comparisons between two groups; *P* < 0.05 was considered statistically significant.

## SUPPLEMENTARY MATERIALS FIGURES AND TABLES




