# Supplementary material for: Model-based unsupervised learning informs metformin-induced cell-migration inhibition through an AMPK-independent mechanism in breast cancer
Source: Oncotarget. 2017 Mar 10;8(16):27199–215. doi: 10.18632/oncotarget.16109 (PMC5432329; doi:10.18632/oncotarget.16109)
Supplement: Supplementary file 1 [file oncotarget-08-27199-s001.pdf]

Model-based unsupervised learning informs metformin-induced cell-migration inhibition through an AMPK-independent mechanism in breast cancer

Supplementary Materials

Supplementary Section 1

Cluster validation using distance-based hierarchical methods

In order to determine if these clusters were recognizable using a distance-based method, we chose two hierarchical clustering methods that use Euclidean distances to find the clusters in metformin-treated cells. *Agglomerative hierarchical clustering* takes a bottom-up approach by iteratively joining the closest cells; *divisive hierarchical clustering* takes a top-down approach by first forming clusters with maximized intercluster distance and breaking them down until intercluster distance is minimized. We tested baseline and metformin-treated cells with both agglomerative hierarchical clustering and divisive hierarchical clustering methods, each of which uses a distance matrix of pairwise Euclidean distances between cells as input. Both hierarchical methods found three clusters in metformin-treated cells, with one cluster comprising the same cells as M2, and with the difference in other clusters being that one cell from M1 was added to M3, with the rest remaining the same. Thus, we were able to establish consistency among the clustering results using different approaches that measured statistical distances between cells (where MiMoSA uses distribution-based distances and hierarchical clustering uses Euclidean distances). Because of the consistent clustering results and high statistical significance in the expression levels of the 230 genes in cluster M2 in comparison with all other clusters, we chose this set of 230 genes for further analyses.

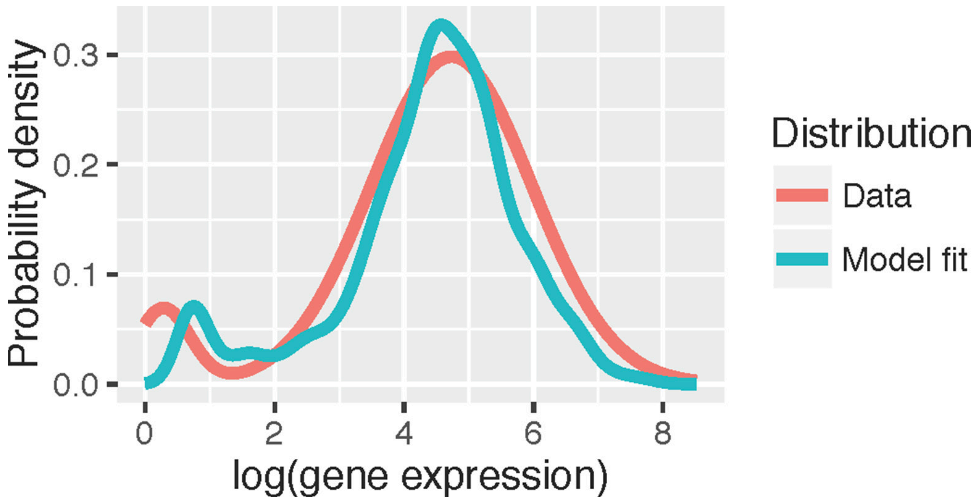

Supplementary Figure 1: The probability density function (PDF) of gene expression within a cell, and the model-fit for the expressions under a Gaussian mixture model with exponential distributions. From a statistical perspective, we tested the samples drawn from the two distributions using the Mann-Whitney *U*-test and Kolmogorov-Smirnov test (KS-test), and the null hypothesis (samples drawn from the identical distribution) was accepted with *p*-value of 0.74 (0.5869 for KS-test) being greater than significance level of 0.05.

Supplementary Table 1: List of pathways significantly enriched in the 230-gene set.

See Supplementary_Table_1

Supplementary Table 2: The 230 differentially expressed genes (bolded and highlighted are 24 genes with less literature evidence in metformin response)

ATP5F1	PSMD6	PBK	TRIAP1	ATP5A1
ATP6V0B	RPL35A	SNHG6	VPS29	IER3IP1
C1orf31	UBA3	UBE2V2	PCID2	NDUFV2
CCT3	UQCRC1	VDAC3	SAP18	PMAIP1
**CDC42**	DANCR	EDF1	TPT1	RPL17
CNIH4	H2AFZ	TOMM5	C14orf2	TXNL1
DDAH1	MRPS18C	ATP5C1	CINP	**AP2S1**
DNTTIP2	**PAICS**	GDI2	**DAD1**	BST2
GAS5	RPL34	GSTO1	JKAMP	C19orf53
NDUFS5	ARRDC3	NDUFB8	NEDD8	COX6B1
NME7	BRIX1	SFTA1P	NPC2	EMP3
**PARK7**	CDK7	VDAC2	PSMA6	FXYD5
**PPT1**	CSNK1A1	ZWINT	PSMC1	**GPI**
RHOC	MRPL36	ATP5L	**RPL36AL**	NDUFA13
RPS8	MTRNR2L2	BANF1	SLIRP	NUDT19
SDHB	NDUFS4	C11orf10	C15orf23	PDCD5
**SSR2**	RPL37	CFL1	**ETFA**	PSMD8
TAGLN2	TAF9	CLNS1A	SCG5	RPL36
UBE2T	VDAC1	CWC15	**SRP14**	RPS28
YBX1	CENPW	FAU	TMEM85	SNRPD2
ZBTB8OS	CLIC1	FTH1	**APRT**	**TECR**
ZMYM6NB	COX7A2	GSTP1	ARL6IP1	UBL5
CCT4	ECI2	**MRPL48**	COX4I1	UCA1
COX5B	EEF1E1	POLR2G	MT1E	AURKA
COX7A2L	GTF3C6	POLR2L	MT2A	DYNLRB1
DPY30	HIST1H1C	PPME1	NQO1	FKBP1A
FARSB	MNF1	SLC35F2	NUTF2	RIN2
HAT1	MRPL18	**TALDO1**	RPS15A	ROMO1
LINC00152	PAK1IP1	TIMM10	RPS2	SRSF6
MRPL33	RPL10A	TMEM179B	TCEB2	YWHAB
NCL	RPL7L1	TMX2	TNFRSF12A	CSTB
OST4	RPS10	TRMT112	UQCRC2	**EIF3D**
PTRHD1	TMEM14C	ARPC3	ANAPC11	EIF3L
RNF181	ARPC1A	ATP5B	ATP5H	NDUFA6
**RRM2**	CHCHD2	CCT2	COPS3	RBX1
SF3B14	CHCHD3	CD63	H3F3B	TOMM22
SSB	MRPS17	**COX14**	KPNA2	TXN2
TXNDC9	**NAMPT**	DYNLL1	LOC100507246	UQCR10
VAMP8	SBDS	EMG1	LSM12	ACOT9
YWHAQ	SHFM1	FAM216A	MRPS7	ATP6AP2
ARPC4	DCAF13	GLTP	**NME1**	EBP
C3orf78	LACTB2	NDUFA12	PFN1	NDUFA1
CCDC72	MRPL13	**PTGES3**	PSMB6	**PRPS2**
**IMPDH2**	**MRPS28**	RPS26	SKA2	RPL10
LSM3	NDUFB9	SLC25A3	SUMO2	RPS4X
POLR2H	PABPC1	TMEM106C	TUBG1	TIMP1
