# Supplementary material for: Model-based unsupervised learning informs metformin-induced cell-migration inhibition through an AMPK-independent mechanism in breast cancer
Source: Oncotarget. 2017 Mar 10;8(16):27199–215. doi: 10.18632/oncotarget.16109 (PMC5432329; doi:10.18632/oncotarget.16109)
Supplement: Supplementary file 2 [file oncotarget-08-27199-s002.docx]

| Supplementary Table 1: List of pathways significantly enriched in the 230-gene set | | | | | | |
| --- | --- | --- | --- | --- | --- | --- |
| Gene set | Ratio of proteins in gene set | Num. of proteins in gene set | Proteins from network | p-value | FDR | Nodes and linkers |
| Metabolic pathways(K) | 0.1244 | 1214 | 39 | 9.61E-30 | 1.35E-28 | COX5B,UQCR10,TECR,NME1,ATP5C1,PRPS2,TALDO1,PPT1,ATP6V0B,COX6B1,NDUFV2,UQCRC2,PTGES3,NAMPT,UQCRC1,ATP5B,NDUFS5,NDUFS4,ATP5L,ATP5H,IMPDH2,COX4I1,NDUFA13,NDUFA12,RRM2,POLR2H,POLR2G,POLR2L,NDUFB8,NDUFB9,DAD1,NDUFA6,ATP5F1,NDUFA1,APRT,SDHB,GPI,ATP5A1,PAICS |
| Parkinson’s disease(K) | 0.0147 | 143 | 27 | 6.57E-22 | 4.60E-21 | COX5B,UQCR10,ATP5C1,COX6B1,VDAC2,VDAC3,VDAC1,NDUFV2,UQCRC2,UQCRC1,ATP5B,NDUFS5,NDUFS4,ATP5H,COX4I1,NDUFA13,NDUFA12,NDUFB8,NDUFB9,COX7A2L,COX7A2,NDUFA6,ATP5F1,NDUFA1,PARK7,SDHB,ATP5A1 |
| Oxidative phosphorylation(K) | 0.0136 | 133 | 25 | 3.81E-21 | 1.78E-20 | COX5B,UQCR10,ATP5C1,ATP6V0B,COX6B1,NDUFV2,UQCRC2,UQCRC1,ATP5B,NDUFS5,NDUFS4,ATP5L,ATP5H,COX4I1,NDUFA13,NDUFA12,NDUFB8,NDUFB9,COX7A2L,COX7A2,NDUFA6,ATP5F1,NDUFA1,SDHB,ATP5A1 |
| The citric acid (TCA) cycle and respiratory electron transport(R) | 0.0148 | 144 | 25 | 2.10E-19 | 7.38E-19 | COX5B,UQCR10,ATP5C1,COX6B1,NDUFV2,UQCRC2,COX14,UQCRC1,ATP5B,NDUFS5,NDUFS4,ATP5L,ATP5H,COX4I1,NDUFA13,NDUFA12,NDUFB8,NDUFB9,COX7A2L,ETFA,NDUFA6,ATP5F1,NDUFA1,SDHB,ATP5A1 |
| Ribosome(K) | 0.0138 | 135 | 23 | 1.96E-18 | 5.49E-18 | RPL17,MRPL36,FAU,RPL10,MRPL33,RPL36AL,RPL35A,MRPS18C,RPS10,MRPS17,RPS26,RPS28,RPL10A,RPS8,RPS2,MRPS7,RPS4X,RPL36,RPS15A,RPL37,MRPL13,RPL34,MRPL18 |
| Huntington’s disease(K) | 0.0198 | 193 | 30 | 4.40E-18 | 1.03E-17 | COX5B,UQCR10,ATP5C1,COX6B1,VDAC2,VDAC3,VDAC1,NDUFV2,UQCRC2,UQCRC1,ATP5B,AP2S1,NDUFS5,NDUFS4,ATP5H,COX4I1,NDUFA13,NDUFA12,POLR2H,POLR2G,POLR2L,NDUFB8,NDUFB9,COX7A2L,COX7A2,NDUFA6,ATP5F1,NDUFA1,SDHB,ATP5A1 |
| Eukaryotic Translation Termination(R) | 0.0086 | 84 | 15 | 8.83E-16 | 1.77E-15 | RPL17,FAU,RPL10,RPL35A,RPS10,RPS26,RPS28,RPL10A,RPS8,RPS2,RPS4X,RPL36,RPS15A,RPL37,RPL34 |
| SRP-dependent cotranslational protein targeting to membrane(R) | 0.0108 | 105 | 17 | 2.46E-15 | 3.90E-15 | SRP14,RPL17,FAU,RPL10,RPL35A,RPS10,RPS26,RPS28,RPL10A,RPS8,RPS2,RPS4X,RPL36,RPS15A,RPL37,RPL34,SSR2 |
| Eukaryotic Translation Elongation(R) | 0.0089 | 87 | 15 | 2.51E-15 | 3.90E-15 | RPL17,FAU,RPL10,RPL35A,RPS10,RPS26,RPS28,RPL10A,RPS8,RPS2,RPS4X,RPL36,RPS15A,RPL37,RPL34 |
| Eukaryotic Translation Initiation(R) | 0.0115 | 112 | 17 | 2.05E-14 | 2.87E-14 | RPL17,FAU,RPL10,RPL35A,RPS10,EIF3D,RPS26,RPS28,RPL10A,PABPC1,RPS8,RPS2,RPS4X,RPL36,RPS15A,RPL37,RPL34 |
| Alzheimer’s disease(K) | 0.0172 | 168 | 23 | 2.87E-14 | 3.65E-14 | COX5B,UQCR10,ATP5C1,COX6B1,NDUFV2,UQCRC2,UQCRC1,ATP5B,NDUFS5,NDUFS4,ATP5H,COX4I1,NDUFA13,NDUFA12,NDUFB8,NDUFB9,COX7A2L,COX7A2,NDUFA6,ATP5F1,NDUFA1,SDHB,ATP5A1 |
| Nonsense-Mediated Decay (NMD)(R) | 0.0109 | 106 | 16 | 5.80E-14 | 6.76E-14 | RPL17,FAU,RPL10,RPL35A,RPS10,RPS26,RPS28,RPL10A,PABPC1,RPS8,RPS2,RPS4X,RPL36,RPS15A,RPL37,RPL34 |
| Non-alcoholic fatty liver disease (NAFLD)(K) | 0.0155 | 151 | 19 | 3.72E-12 | 4.00E-12 | COX5B,UQCR10,COX6B1,NDUFV2,UQCRC2,UQCRC1,CDC42,NDUFS5,NDUFS4,COX4I1,NDUFA13,NDUFA12,NDUFB8,NDUFB9,COX7A2L,COX7A2,NDUFA6,NDUFA1,SDHB |
| Mitochondrial translation(R) | 0.0091 | 89 | 9 | 1.41E-07 | 1.41E-07 | MRPL36,MRPL33,MRPS18C,MRPL48,MRPS17,MRPS28,MRPS7,MRPL13,MRPL18 |
